# 138 Evaluation Of The Use of Antibiotic Locks For the Treatment and Prevention of CLABSI

**DOI:** 10.1017/ash.2026.10546

**Published:** 2026-06-23

**Authors:** Amit Vahia, Meenal Malviya, Alexander Cardwell, Leonard Johnson, Susan Szpunar

**Affiliations:** 1 Henry Ford St. John Hospital; 2 Henry Ford St John Hospital

## Abstract

**Background:** Clostridioides difficile infection (CDI) has been diagnosed with a variety of test methods that vary in their sensitivity and specificity. Because of concerns over potential overdiagnosis of clinical CDI, a single step method was adopted in May 2023 with glutamate dehydrogenase (GDH) and toxin enzyme immunoassay (EIA) with nucleic acid amplification test (NAAT) only to be ordered by clinicians who had ongoing clinical suspicion of CDI despite indeterminate test results. We aimed to describe the physician response to indeterminate test results, evaluate the percentage of people who had re-admission within 90 days for all-cause mortality and those with re-admission for diarrhea Methods: A multicenter historical cohort study was conducted among patients who were found to have GDH+/toxin- C. difficile results from 6/1/2023 to 12/31/24. Data were collected by retrospective chart review on the initial hospitalization and subsequent episodes of CDI in the following 90 days based on receipt of treatment for the initial episode of GDH+/toxin-. Data were analyzed using Student’s t-test, the Mann-Whitney U test, and the chi-squared test, using SPSS v. 31.0. **Results:** Of 382 patients, 380 had complete information on treatment history. Of these patients, 25% were treated (95/380). Among 285 patients with indeterminate C.difficile tests who were untreated and had re-admission in 90 days for diarrhea, only 2.1% were diagnosed with C.difficile colitis on the follow up admission. Patients who received vancomycin alone had the highest percentage of re-admission among other treatment options. Patients with chronic steroid use (p=0.05), other immunosuppressive agents (p=0.02) and with inflammatory bowel disease (IBD) (p=0.002) were significantly more likely to be readmitted for diarrhea within 90 days. **Conclusion:** A physician-based approach reduces overtreatment of indeterminate C. difficile results using a single step reporting method with only a small number of patients readmitted with CDI within 90 days.

